# Search and foraging behaviors from movement data: A comparison of methods

**DOI:** 10.1002/ece3.3593

**Published:** 2017-11-23

**Authors:** Ashley Bennison, Stuart Bearhop, Thomas W. Bodey, Stephen C. Votier, W. James Grecian, Ewan D. Wakefield, Keith C. Hamer, Mark Jessopp

**Affiliations:** ^1^ MaREI Centre for Marine and Renewable Energy Environmental Research Institute University College Cork Cork Ireland; ^2^ School of Biological Earth, and Environmental Sciences (BEES) University College Cork Cork Ireland; ^3^ Centre for Ecology & Conservation University of Exeter Penryn UK; ^4^ Environment & Sustainability Institute University of Exeter Penryn UK; ^5^ Sea Mammal Research Unit Scottish Oceans Institute University of St Andrews St Andrews, Fife Scotland; ^6^ Institute of Biodiversity, Animal Health and Comparative Medicine College of Medical, Veterinary, and Life Sciences University of Glasgow Glasgow Scotland; ^7^ Faculty of Biological Sciences School of Biology University of Leeds Leeds UK

**Keywords:** behavior, first passage time, hidden Markov models, kernel density, *k*‐means, machine learning, movement, state‐space models, telemetry

## Abstract

Search behavior is often used as a proxy for foraging effort within studies of animal movement, despite it being only one part of the foraging process, which also includes prey capture. While methods for validating prey capture exist, many studies rely solely on behavioral annotation of animal movement data to identify search and infer prey capture attempts. However, the degree to which search correlates with prey capture is largely untested. This study applied seven behavioral annotation methods to identify search behavior from GPS tracks of northern gannets (*Morus bassanus*), and compared outputs to the occurrence of dives recorded by simultaneously deployed time–depth recorders. We tested how behavioral annotation methods vary in their ability to identify search behavior leading to dive events. There was considerable variation in the number of dives occurring within search areas across methods. Hidden Markov models proved to be the most successful, with 81% of all dives occurring within areas identified as search. *k*‐Means clustering and first passage time had the highest rates of dives occurring outside identified search behavior. First passage time and hidden Markov models had the lowest rates of false positives, identifying fewer search areas with no dives. All behavioral annotation methods had advantages and drawbacks in terms of the complexity of analysis and ability to reflect prey capture events while minimizing the number of false positives and false negatives. We used these results, with consideration of analytical difficulty, to provide advice on the most appropriate methods for use where prey capture behavior is not available. This study highlights a need to critically assess and carefully choose a behavioral annotation method suitable for the research question being addressed, or resulting species management frameworks established.

## 1 INTRODUCTION

Movement is major part of a species’ ecology. The underlying processes driving the movement of individuals and populations are studied widely; however, it is often unfeasible to directly observe animals through constant effort. As a result, movement studies have focussed on remote detection of animals through technologies such as GPS and satellite tracking. The development, miniaturization, and reduction of cost in remote tracking technologies have enabled its widespread use in ecological studies (Cagnacci, Boitani, Powell, & Boyce, 2010). Remote tracking enables behaviors to be inferred from an animals’ trajectory (Buchin, Driemel, Kreveld, & Sacristán, 2010), and has led to rapid advances in the understanding of species’ ecology (Nathan et al., 2008).

While movement patterns are often used to distinguish active phases from rest, or search behavior from traveling (van Beest & Milner, 2013; Dzialak, Olson, Webb, Harju, & Winstead, 2015), identifying these behavioral states typically relies on more complicated modeling procedures to detect potential underlying mechanisms within behavior identification (Jonsen, Myers, & James, 2006; Kerk et al., 2015). Considerable progress has been made in developing methods that can categorize behaviors based on simple movement metrics (Edelhoff, Signer, & Balkenhol, 2016). These methods commonly identify multiple states and ascribe these to predefined behaviors such as search, rest, or travel (Evans, Dall, Bolton, Owen, & Votier, 2015; Guilford et al., 2008; Hamer, Phillips, Wanless, Harris, & Wood, 2000; King, Glahn, & Andrews, 1995; Palmer & Woinarski, 1999; Shepard, Ross, & Portugal, 2016; Weimerskirch et al., 2006). However, Gurarie et al. (2016) argued for closer and more detailed exploratory analysis of movement data to prevent mis‐specification of behavior, suggesting that the strengths of particular methods need to be more carefully considered so they are suitably attuned to the specific questions being asked by researchers.

Within conservation management, there is an increasing reliance on identifying space use by species of conservation concern (Allen & Singh, 2016). For example, within the marine environment, foraging areas could be considered for the protection and management of seabird species (Lascelles et al., 2016). The use of these approaches may contribute to the establishment of conservation measures including designation of marine protected areas (Grüss, Kaplan, Guénette, Roberts, & Botsford, 2011). Foraging activity is a key component in an animal's time and energy budget, and it is well established that animals in environments with patchy resources must engage in search behavior to optimize their foraging effort in terms of maximizing prey encounters (MacArthur & Pianka, 1966). Therefore, foraging can be considered a two‐part system, containing both search and prey capture attempts (Charnov, 1976). Understanding the interaction between search and prey capture is a key component in optimal foraging theory (Pyke, 1984). For example, while there has been much work identifying area‐restricted search (Knell & Codling, 2012), there is little information on the relationship between search and prey capture. Validation of search behavior is difficult particularly in animals where direct observation is challenging, such as those in many biotelemetry studies. Many movement studies use path segmentation techniques to detect search behavior; however, many of these are unvalidated estimates of search due to the lack of a second data stream for ground‐truthing. Validation of prey capture attempts has been achieved using animal‐borne cameras (Bicknell, Godley, Sheehan, Votier, & Witt, 2016; Moll, Millspaugh, Beringer, Sartwell, & He, 2007), time–depth recorders (Dean et al., 2012; Shoji et al., 2015; Tinker, Costa, Estes, & Wieringa, 2007), stomach loggers (Weimerskirch, Gault, & Cherel, 2005), and accelerometers (Hansen, Lascelles, Keene, Adams, & Thomson, 2007; Sato et al., 2007) among others. However, many of these technologies are either expensive resulting in small sample sizes or are too large to deploy on animals in combination with location loggers without significant adverse impacts (Barron, Brawn, & Weatherhead, 2010; Hammerschlag, Gallagher, & Lazarre, 2011; Vandenabeele, Shepard, Grogan, & Wilson, 2012). As a result, many studies still rely on the sole use of location data and path segmentation approaches to identify behavior. The determination of behavior from movement data is an active area of research and the subject of many reviews (Allen, Metaxas, & Snelgrove, 2017; Edelhoff et al., 2016; Hays et al., 2016; Jacoby, Brooks, Croft, & Sims, 2012). There are several different methods for undertaking behavioral annotation or detecting important areas of high use by animals. Frequently used are movement pattern description and process identification. Methods based around movement pattern description are often aimed at trying to split between different behavioral periods or to locate changes in behavior (Edelhoff et al., 2016). Process identification aims to take things a step further and concentrates on methods that are focussed toward being able to describe the underlying processes, whether extrinsic or intrinsic, and describe how these inform behavior.

Northern gannets (*Morus bassanus)*, hereafter gannets, are a well‐studied species that occur principally in the temperate shelf seas of the North Atlantic during the breeding season. Gannets are visual predators (Cronin, 2012) and undertake plunge‐diving from height, entering the water at speeds of up to 24 m/s (Chang et al., 2016). Prior to diving, gannets typically slow their flight and increase their path sinuosity (Wakefield et al., 2013; Bodey et al., 2014; Patrick et al., 2014; Warwick‐Evans et al., 2015). The relationship between slow speed during search and prey capture attempts has been established both theoretically (Bartoń & Hovestadt, 2013; Benhamou, 2004) and empirically in a variety of mobile marine and terrestrial species (Anderson & Lindzey, 2003; Byrne & Chamberlain, 2012; Edwards, Quinn, Wakefield, Miller, & Thompson, 2013; McCarthy, Heppell, Royer, Freitas, & Dellinger, 2010; Towner et al., 2016; Wakefield et al., 2013; Williams et al., 2014). Such changes in movement and clearly identifiable prey capture attempts in the form of dives (Cleasby et al., 2015; Garthe, Benvenuti, & Montevecchi, 2000), as well as their ability to carry multiple devices and ease of recapture, make gannets a suitable model species to explore the ability of movement‐based analysis to identify search behavior and prey capture attempts.

In this study, we apply and compare seven methodologies covering movement pattern description and process identification, to predict search behavior in gannets using GPS location data. If search behavior is a precursor to prey capture attempts, dives will occur primarily within areas identified as search. With consideration given to opportunistic foraging, we hypothesize that more successful methods of search classification will contain more true positives (dive events occurring within identified search), fewer false positives (search containing no dives), and fewer false negatives (dives occurring outside identified search behavior). Using this framework, we will also provide recommendations on the appropriate use of methodological approaches.

## 2 MATERIALS AND METHODS

### 2.1 Data collection

Breeding adults at two island colonies, Great Saltee, Co. Wexford, Ireland (52.11286, −6.62189) and Bass Rock, Scotland, UK (56.07672, −2.64139), were tracked while attending 2 to 7‐week‐old chicks over a 38‐day period from late June to early August 2011. Nine birds at Great Saltee and eight birds at Bass Rock were caught using a metal crook or wire noose fitted to a 4 to 6‐m pole and fitted with GPS loggers coupled with time–depth recorders (TDRs). GPS loggers (i‐gotU GT‐200, Mobile Action Technology Inc., Taipei, Taiwan, 37 g), sealed in heatshrink plastic, recorded locations every 2 min. CEFAS G5 TDRs (CEFAS Technology, Lowestoft, UK, 2.5 g) were deployed using the fast‐log dive sensor at 4 Hz and used to identify dive events based on a 1 m depth threshold being exceeded, hereafter TDR dives. This was to ensure dives reflected prey capture attempts (median dive depth of 4.6 m in plunge‐diving gannets and 8 m when pursuit diving (Garthe et al., 2000) rather than other surface‐related activities such as resting, washing, or preening. Devices were attached following (Grémillet et al., 2004), and involved affixing loggers ventrally to 2–4 central tail feathers using strips of waterproof Tesa© tape. Total instrument mass was ≤2% of body mass, below the maximum recommended for seabird biologging studies (Phillips, Xavier, & Croxall, 2003), and tag position was considered to minimally impede gannets aerodynamically or hydrodynamically (Vandenabeele et al., 2012). Deployment and retrieval handling times were approximately 10 min.

### 2.2 Data processing

GPS tracks were processed using the *AdehabitatLT* package (Calenge, 2011) in the R statistical Framework. Location data were transformed into Cartesian coordinates using a Universal Transverse Mercator (UTM) 30N projection before calculating step length and turning angles. Although GPS tags were programmed to take locations every 2 min, if there was no available GPS signal (because a bird was diving for example), locations may not have been exactly two minutes apart, and so tracks were standardized through linear interpolation to a two‐minute interval. Speed, step length, turning angle, and distance from colony were calculated for every point along a bird's track. Points within 5 km of the colony were removed to avoid potential locations associated with colony rafting and bathing (Carter et al., 2016), as were those occurring at night (between civil sunset and sunrise) because gannets are visual diurnal foragers (Nelson, 2002). TDR dives were split into dive events and produced a single timestamp point representing the start of any dive event over 1 m for appending to tracks following behavioral classification.

We applied a suite of methods commonly used to identify searching or infer foraging behaviors from movement data, summarized in Table 1. The methods are not considered exhaustive, but represent a range of approaches covering movement pattern description and process identification (Edelhoff et al., 2016). Movement pattern description approaches include kernel density, first passage time (FPT), and speed/tortuosity thresholds, while process identification techniques applied covered *k*‐means clustering and two state‐space models, hidden Markov models (HMM) and effective maximization binary clustering (EMbC). The two forms of state‐space models were used to represent diverging classes of state‐space model; maximum likelihood methods (EMbC), and Bayesian Monte Carlo methods (HMM) (Patterson, Thomas, Wilcox, Ovaskainen, & Matthiopoulos, 2008). While not predicting/identifying search behavior directly, we also applied machine learning (generalized boosted regression models) to predict dives from track metrics rather than search behavior. We followed the standard methodology for each technique outlined in the published literature, and provide references for detailed guidance on applying each approach.

**Table 1:** Summary of common methodological approaches to identifying search and foraging behavior in movement data. While all methods require validation data to assess how well the method works, it is not necessarily required to implement the method

Method	Analysis complexity	Requires validation data	Suitable for investigating relationships with environmental variables	Immediately applicable to other species/locations
Machine learning	High & large data requirement	Yes	Yes	No
*k*‐Means	Low	No	Yes	Yes
Thresholds	Medium	Yes	Yes	No
FPT	Medium	No	Yes	Yes
HMM	Medium	No^a^	Yes	Yes
Kernel density	Low	No	Dependent on scale	Yes
EMbC	Low	No	Yes	Yes

a HMM do not require validation data in this context, but can employ if desired.

**Figure 1:**
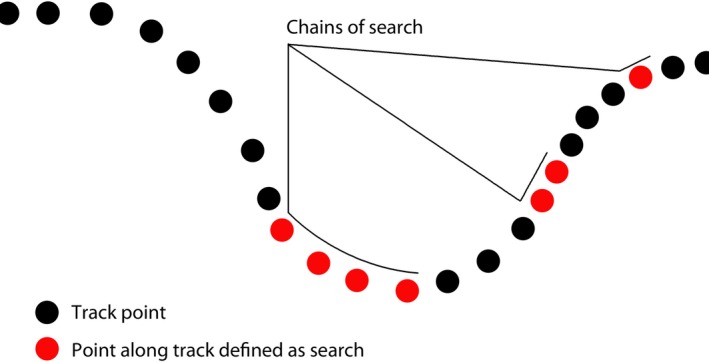
Conceptual diagram of locations through time identifying points of search behavior within the series that reveal search chains of differing lengths

Methods of predicting search behavior routinely identify chains of search in successive locations. Chains can be a single point in length or may include multiple consecutive points along a movement track (see Figure 1). Given that in gannets, individual prey capture attempts (dives) occur at discrete locations/times, we extracted metrics of dives occurring within search, dives outside of search, and search containing no dive. Data from the two colonies were processed independently to account for potential differences in movement metrics associated with differences in local habitat and prey availability.

### 2.3 Kernel density

Time in space is considered to be a good proxy for foraging effort (Warwick‐Evans et al., 2015). GPS locations (excluding locations within 5 km of the colony and locations at night) were used to estimate kernel densities in ArcMap 10.2, which uses a kernel smoothing function based on the quartic kernel function by Silverman (1986), and had a bandwidth/search distance of 10 km. This was used to create a kernel density square grid with sides of 10 km. The method produces a 10 km^2^ grid with relative intensity of both TDR dives and GPS tracks. Dutilleul's modified spatial *t* test (Dutilleul, Clifford, Richardson, & Hemon, 1993) was used to determine the spatial correlation between the intensity of dives and intensity of tracks, accounting for spatial autocorrelation in the data.

### 2.4 First passage time

First passage time (FPT) analysis was undertaken following Fauchald and Tveraa (2003). Although tracks were rediscretized in time for all other analysis, FPT requires tracks to be redistributed in space to account for changes in bird speed, and so tracks were redistributed using linear interpolation to 500‐m distances. Analysis was undertaken using the *AdehabitatLT* package in R (Calenge, 2011). Based on the behavioral response ranges reported by Bodey et al. (2014) for gannets, circles of radii ranging from 50 m to 12,000 m were used to construct first passage time values. The maximum log‐variance of first passage time values was then used to determine appropriate search radii for each individual bird. The slowest sextile of passage times was considered to be relatively higher intensity search behavior as used by Nordstrom, Battaile, Cotte, and Trites (2013), and also indicated in work by Hamer et al. (2009) following Fauchald and Tveraa (2003). Search radii were used to create an amalgamated area of search along an individual bird's track, with GPS points along this track treated as “search” points. Although FPT can be used to determine nested levels of area‐restricted search (Hamer et al., 2009), we have considered only the highest levels of search behavior to maximize the number of dives potentially occurring within search.

### 2.5 *k*‐Means clustering

*k*‐Means clustering is a method of vector quantization that aims to partition *n* observations into *k* clusters, and has been used to cluster data points consistent with different behaviors (Jain, 2010). *k*‐Means clustering was undertaken using the MacQueen algorithm (MacQueen, 1967) on step length and turning angle between successive GPS locations. The optimum number of clusters was determined using the “elbow method” where the percentage of variance explained (the ratio of the between‐group variance to the total variance) is plotted as a function of the number of clusters and the point where addition of further clusters results in only marginal increases in explained variance (Ketchen & Shook, 1996). This resulted in three clusters, and these were then assigned behavioral states based on logical differences between the means of variables in each group. The cluster with largest step length and smallest tortuosity was defined as travel, short step length and intermediate tortuosity were considered consistent with rest, and intermediate step length and high tortuosity were considered consistent with search behavior following Zhang, O'Reilly, Perry, Taylor, and Dennis (2015).

### 2.6 Speed–tortuosity thresholds

Speed–tortuosity thresholds from Wakefield et al. (2013) were applied to the data. These were developed based on prior knowledge of gannet foraging behavior and an iterative examination of plausible thresholds of movement indices from those initially suggested by Grémillet et al. (2004). Thresholds suggested by Wakefield et al. (2013) were applied as they were based on data from tracked gannets from a range of colonies, including the data analyzed in this study. Successive GPS locations were considered to represent search if they met any one of three conditions:

1. Tortuosity <0.9 and speed >1 m/s
2. Speed >1.5 m/s and <9 m/s
3. Tortuosity ≥0.9 and acceleration <−4 m/s^2^

Speed and acceleration were calculated between *L*_−1_ and *L*_0_ where *L*_0_ is the focal point, while tortuosity is the ratio of the straight line to along the track distance between *L*_−4_ and *L*_4_. Criteria were defined based on GPS and TDR data from Bass Rock deployments used in this study and are therefore created from a priori information.

### 2.7 Hidden Markov Models

Hidden Markov Models (HMM) are an example of state‐space modeling, where models are formed of two parts, an observable series and a nonobservable state sequence (Langrock et al., 2012). The observable series, in this context, take the form of GPS relocations with consequential step length and turning angle, while the nonobservable are behavioral states. HMM use a time series to determine what denotes the underlying states and the changes between them. The application of state‐switching models to movement data allows behavioral modes to be examined, while considering the high degree of autocorrelation present in telemetry data (Patterson et al., 2008). When the observational error is low, hidden Markov models offer a more tractable approach to discretize behavioral modes from telemetry data than Bayesian approaches (Langrock et al., 2012). Using the R package *moveHMM* (Michelot, Langrock, & Patterson, 2016), the movement of each individual along a foraging trip was classified into one of three underlying states by characterization of the distributions of step lengths and turning angles between consecutive locations. A three‐state model was a better fit to the data than a two‐state model, and is consistent with previous work describing gannet movement (Bodey et al., 2014) as well as the identification of three states in EMbC and *k*‐means clustering approaches within this study. Model iterations successfully converged to three states suggesting a good fit to the data. A gamma distribution was used to describe the step lengths, a von Mises distribution described the turning angles, and the Viterbi algorithm was used to estimate the most likely sequence of movement states to have generated the observations (Zucchini, MacDonald, & Langrock, 2016).

### 2.8 Expectation–maximization binary clustering

Expectation–maximization binary clustering (EMbC) protocols are an unsupervised, multivariate example of a state‐space modeling framework that can be used for behavioral annotation of movement trajectories, including search behavior (see Garriga, Palmer, Oltra, and Bartumeus (2015)). EMbC has been designed to be a simple method of analyzing movement data based on the geometry alone, and can behaviorally annotate movement data with minimal supervision. EMbC is a relatively modern technique that is gaining traction within movement ecology. It has previously been used in a variety of movement studies, including exploring behavioral differences between distinct populations of the red‐footed booby (Mendez et al., 2017) and coupling energy budgets with behavioral patterns under an optimal foraging framework (Louzao, Wiegand, Bartumeus, & Weimerskirch, 2014). Analysis was undertaken using the *EMbC* package in R (Garriga & Bartumeus, 2016), using calculated velocities and turning angles to infer behavioral classifications.

### 2.9 Machine learning

While the methods outlined above all identify search behavior, machine learning models are trained to specifically identify prey capture/dive events based on track metrics. Analysis was undertaken using the *Caret* package in R (Kuhn, 2008) using generalized boosted regression models to account for zero‐inflation (Elith, Leathwick, & Hastie, 2008). Models were built using step length, speed, turning angle, hour of day, and tortuosity. Models were trained using 75% of the linked GPS/TDR dive data, with the remaining 25% of data kept for validation of predictions, and underwent cross‐validation 500 times during the training procedure. By combining all individual animal's data in this manner, we ensure that any intra‐individual variation is accounted for in the modeling process. Receiver operator curves (ROCs) were calculated (Fielding & Bell, 1997) to determine the model of best fit at each colony.

### 2.10 Comparison of methods using TDR dives

In order to compare the predictive power of the seven methods outlined above in predicting areas in which dives occurred, TDR dive events were linked to GPS coordinates by matching the time/date stamps of both datasets for each individually tracked bird. To compare how well the methods capture dive events within areas of search, the proportion of dives within identified areas of search (true positive) as well as the number of search chains containing no dives (false positive) was calculated for FPT, *k*‐means, thresholds, HMM, and EmbC. The correlation between kernel densities of GPS tracks and TDR dives was assessed using a Dutilleul's modified spatial *t* test (Dutilleul et al., 1993). This analysis provides a correlation coefficient across the spatial extent of the tracked data to determine how well the two datasets correlate while accounting for spatial autocorrelation. Model performance for machine learning was assessed using kappa values, a measure of variability explained by the model akin to *R* ^2^ values, where 0 is equal to no relationship and 1 is equal to a perfect relationship as per Landis and Koch (1977). Further to this, a confusion matrix was calculated by running models on the remaining 25% test data to assess the number of correctly and incorrectly identified dives.

## 3 RESULTS

Nine GPS & TDR combinations were deployed at Great Saltee, resulting in 31,716 locations after standardization to a two‐minute interval. Eight GPS & TDR combinations were deployed at Bass Rock, resulting in 21,208 relocations when standardized. There were a total of 2,830 TDR dives among the tracked birds at Great Saltee and 2,172 at Bass Rock. Examples of maps produced by methods and showing the location of TDR dives can be seen in the Supplementary Materials (see Figs. S1–10).

FPT, *k*‐means, EMbC, thresholds, and HMM all predict search rather than prey capture attempts per se. All methods predicted considerable search effort across the tracking period (Table 2). FPT identified the longest contiguous chains of search behavior (mean 24.74 locations/chain), followed by HMM (mean 8.58 locations/chain), speed and tortuosity thresholds (mean 4.57 locations/chain), and EMbC (mean 2.38 locations/chain). *k*‐Means method identified the most discrete search areas with the shortest chains (mean 3.08 locations/chain). Using Kendall's tau correlation, there was a weak positive correlation between the length of search chains and the number of dives occurring within them (Table 3).

**Table 2:** Comparison of search identification across methods at Great Saltee and Bass Rock with associated TDR dives at each colony. True positives are when a dive occurs within a chain of locations identified as search, false positives are when a chain of locations identified as search does not contain a TDR dive, and false negatives are when a dive occurs outside of areas identified as search, and will include opportunistic foraging events

Method	Great Saltee				Bass Rock			
	No of relocations: 31,716No of dives: 2,830				No of relocations: 21,208No of dives: 2,172			
	Rate of true positives (% dives in search)	Rate of false positives (% search chains with no dive)	Rate of false negatives (% dives outside of search)	Time spent in searching^b^ (% of relocations searching)	Rate of true positives (% dives in search)	Rate of false positives (% search chains with no dive)	Rate of false negatives (% dives outside of search)	Time spent in searching^b^ (% of relocations searching)
FPT	30.59	57.30	69.41	16.68	29.68	46.42	70.32	16.63
*k*‐Means clustering	37.52	74.00	62.48	27.27	21.91	79.92	78.09	19.17
Thresholds	76.81	67.98	23.19	37.15	57.50	63.95	42.50	28.69
HMM	80.81	63.05	19.19	41.53	81.30	56.76	18.70	36.67
EMbC	50.91	73.90	49.09	20.71	46.04	68.61	53.96	27.26
Kernel density	N/A^a^	N/A^a^	N/A^a^	N/A^a^	N/A^a^	N/A^a^	N/A^a^	N/A^a^
Machine learning	N/A^a^	N/A^a^	N/A^a^	N/A^a^	N/A^a^	N/A^a^	N/A^a^	N/A^a^

a Machine learning and kernel density assessed with other metrics due to nature of analysis, see Tables 3, 4, 5.

b Nighttime and locations close to the colony have been omitted. The remaining proportion of relocations is considered to be a combination of rest and travel.

**Table 3:** Kendall's tau correlation between search chain length and number of dives contained within each chain

Method	Correlation (tau)	*p* Value	*Z* statistic
FPT	0.43	<.01	12.67
*k*‐Means clustering	0.30	<.01	21.76
Thresholds	0.45	<.01	33.72
HMM	0.47	<.01	23.79
EMbC	0.39	<.01	31.29

**Figure 2:**
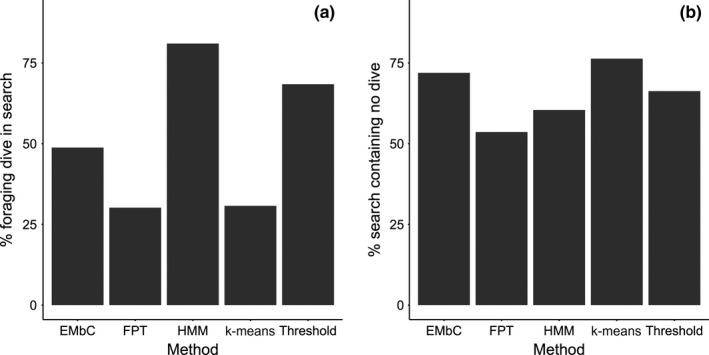
Proportion of (a) TDR dives occurring within ‘search” behavior (true positives) and (b) search chains containing no TDR dives (false positives) using EMbC, FPT, HMM,* k*‐means, and speed–tortuosity thresholds

The performance of behavioral classification methods was assessed by comparing the occurrence of TDR dives inside and outside of predicted search behavior (Table 2). HMM captured the highest proportion of TDR dives (Figure 2a) within search areas, and had the second lowest false‐positive rate (Figure 2b). FPT had the longest identified search chains, but these actually captured the lowest number of dives across all methods (Table 2, Figure 2a). Despite the low true‐positive rates, FPT had the lowest false‐positive rate (Figure 2b). Thresholds and EMbC were comparatively similar in both the rates of true and false positives, while *k*‐means clustering had the lowest true‐positive and highest false‐positive rates of all methods tested.

**Figure 3:**
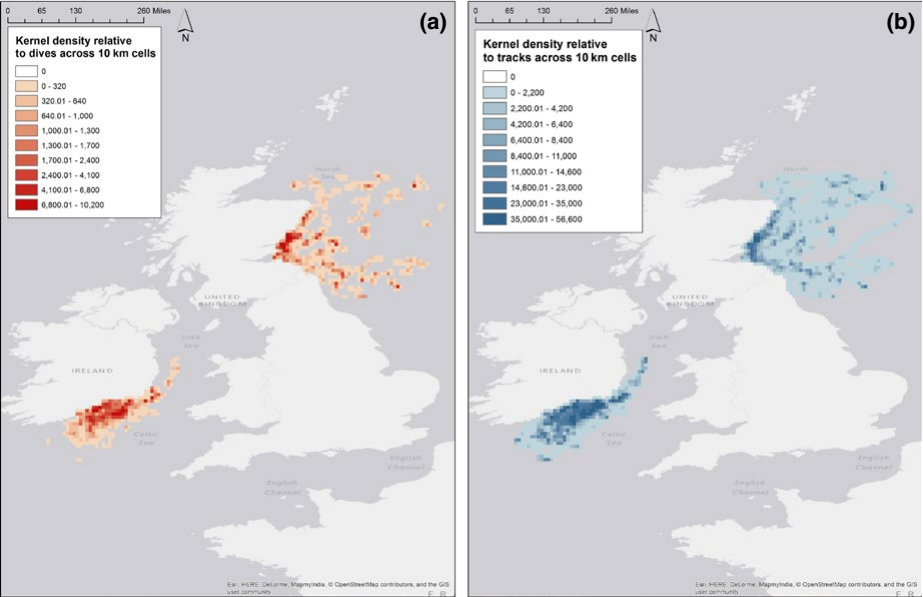
Kernel densities of gannet tracks at both Great Saltee and Bass Rock for (a) dive locations and (b) individual bird tracks. Scale is of relative time in space across the spatial boundary of 10 km throughout the tracking area

Kernel density of GPS locations did not explicitly identify search behavior but identified “hot spots” of foraging corresponding to time spent in each 10 × 10 km grid cell, with a high proportion of time spent in the area surrounding colonies (Figure 3). Dutilleul's modified spatial *t* test demonstrated a good correlation between the spatial distribution of TDR dives and time in space (Table 4), with the better correlation (0.86) at Bass Rock. Machine learning models directly predicted the location of prey capture events. The models trained and tested on their own colony indicated only a fair or slight agreement within the data (following Landis and Koch, 1977) (Table 5). Furthermore, the confusion matrix (Table 6) showed that the predictive power of the models at both colonies was poor, only successfully predicting 22% of dives in the test dataset. When models built in one colony were applied to others, there was a further loss of predictive power, indicating that model structures and movement patterns between colonies are different (Table 4).

**Table 4:** Dutilleul's correlation between kernel densities of all GPS locations and confirmed dive locations

Colony	Correlation	*p* Value	*F* statistic	Degrees of freedom
Great Saltee	0.79	<.01	123.37	69.57
Bass Rock	0.87	<.01	991.88	329.90

**Table 5:** Kappa values for machine learning models where models developed using colony‐specific data are applied at the colony from which training data were taken and at a different colony. Low values for models trained at one colony applied to the other colony suggest very poor model fit

Model trained	Model applied	
	Great Saltee	Bass Rock
Great Saltee	0.2456	−0.0006757
Bass Rock	0.02792	0.1885

**Table 6:** Confusion matrix table totals of predictions made across machine learning models at both Great Saltee and Bass Rock

Predicted result	Reference (true value) in test data set	
	Dive	No dive
Dive	222	258
No dive	779	5,332

## 4 DISCUSSION

Seven methods of classifying search behavior were compared to a validation dataset of TDR dive events in northern gannets to determine their ability to accurately capture the two components of foraging activity—searching and prey encounter/capture. Across methods, the number of prey capture attempts (TDR dives) within search varied considerably, with the highest being captured by hidden Markov models (81%) and the lowest captured by first passage time and *k*‐means clustering (30% and 31%, respectively). While HMM had the highest rate of capture of dive events, it also had one of the lowest rates of false positives, identifying fewer search chains where no dive was recorded. While this was still relatively high (60%), all methods produced high numbers of search chains that contained no TDR dives (range 53%–76%). There was a weak correlation between chain length and number dives within a chain. While prey capture attempts will increase with trip and search duration (Sommerfeld, Kato, Ropert‐Coudert, Garthe, & Hindell, 2013), the weak correlation represents some longer search chains containing relatively few prey capture attempts due to individuals searching over poor‐quality areas, or simply that search does not always result in prey capture attempts. These findings suggests that significant effort is spent in unsuccessful search behavior, consistent with low prey encounter rates associated with foraging on spatially and temporally patchy prey resources (Weimerskirch, 2007).

While the spatial distribution of tracked gannets will encompass a variety of behaviors including foraging, travel, and rest periods, simpler methodologies such as kernel density estimation of track data correlated well with kernel densities of TDR dive events. This supports the assertion that time in area is a good proxy for foraging effort (Grémillet et al. 2004; Warwick‐Evans et al., 2015). However, this approach utilizes larger areas of space beyond movement paths, and so it is not capable of identifying foraging in association with temporally ephemeral events or features that may directly change an animal's movement trajectory. Within more process‐driven approaches, FPT is arguably one of the most ubiquitous methods used to identify foraging areas in both terrestrial and marine systems (Battaile, Nordstrom, Liebsch, & Trites, 2015; Byrne & Chamberlain, 2012; Evans et al., 2015; Hamer et al., 2009; Le Corre, Dussault, & Côté, 2014). FPT captures search behavior across multiple spatial scales and is particularly noted for its ability to detect nested scales of area‐restricted search (Hamer et al., 2009). While we did not investigate nested scales of search, FPT, along with *k*‐means clustering, had the lowest rate of dives occurring within broad areas of identified search. However, in contrast to *k*‐means, FPT had the lowest rate of false positives (search containing no dives), likely as a result of identifying very large, contiguous areas of search. *k*‐Means clustering and FPT had high rates of false negatives, with approximately 70% of all dives occurring outside identified search behavior. A certain amount of opportunistic foraging is anticipated in any wide‐ranging predator (Montevecchi, Benvenuti, Garthe, Davoren, & Fifield, 2009), resulting in dive events occurring outside classical patterns of search movement. However, the high rate of dives occurring outside search as defined by FPT and *k*‐means suggests that either the majority of prey capture attempts occur opportunistically or that the scale of ARS changes spatially, resulting in search behavior associated with dives being missed.

Speed–tortuosity thresholds “captured” 68% of TDR dives within areas identified as search. There is evidence to suggest that humans are more capable than machines at pattern recognition when presented with limited data (Samal & Lyengar, 1992). It is therefore unsurprising that thresholds performed well considering that they were constructed based on prior knowledge of foraging behavior and iterative examination of thresholds against a validation dataset in gannets (Wakefield et al., 2013). The relatively high rates of false positives (66% of search chains containing no TDR dive) were within the spread of values for other methods, highlighting significant effort spent searching for prey interspersed with relatively few prey encounters.

The state‐space modeling framework has been acknowledged as particularly useful in movement ecology (Patterson et al., 2008), and is rapidly expanding within path segmentation techniques (Michelot et al., 2016; Roberts & Rosenthal, 2004). Both the EMbC and HMM approaches model the changes in step length and turning angle through time and space to annotate the trajectory of an animal with behavioral states (Garriga et al., 2016; Michelot et al., 2016). EMbC protocols resulted in shorter search chains that encapsulated 49% of all dive events, while HMM identified longer chains of search that captured the highest number of dives (81%) of any method. While HMM defined the highest number of points as search across all methods, it also had one of the lowest rates of false positives. Less than 20% of dives occurred outside of search. This would be more consistent with opportunistic foraging and provides further empirical evidence of search behavior leading to prey capture attempts (Dias, Granadeiro, & Palmeirim, 2009; Weimerskirch, Pinaud, Pawlowski, & Bost, 2007). The high number of shorter search chains identified by EMbC, coupled with the fact that it is possible to link state transitions to environmental covariates in a HMM framework, suggests that both these methods may also be suitable for or investigating behavioral response to ephemeral environmental cues.

Regional differences in habitat and prey, as well as inter‐ and intraspecific competition are likely to influence the way an animal forages (Huig, Buijs, & Kleyheeg, 2016; Schultz, 1983; Zach & Falls, 1979). To account for this, the colonies were treated independently during analysis. Machine learning did highlight slight differences between colonies in the movement metrics considered to be of most predictive power, suggesting local differences in movement associated with foraging and search. Machine learning was the only method that directly predicted prey capture events rather than search behavior. While the explanatory power of the models was deemed to be satisfactory, the predictive ability of models was poor, only correctly identifying 22% of dives in the test dataset. The success of this method may have been limited by the available sample size. As a powerful tool, machine learning approaches do require large amounts of data, are computationally complex, and require a priori knowledge of dive events to train the model. However, machine learning protocols are still being developed within ecological research, and such data mining remains a challenge for accurate classification (Hochachka et al., 2007).

An interesting consideration throughout the methods presented, here, is the ability to identify multiple behavioral states. HMM, *k*‐means, and EMbC are capable of identifying behavior consistent with rest within the tracking period (typically very low speed and a medium‐to‐high tortuosity values). In this context, kernel density, FPT, speed–tortuosity thresholds, and machine learning did not identify periods of rest. The majority of behavioral annotation relies on the principle of animals slowing down and paths becoming more tortuous when searching (Bartoń & Hovestadt, 2013; Benhamou, 2004). However, slowing down and turning more could also be an indication of rest behavior, especially when considering potential error from closely positioned GPS relocations (Hurford, 2009; Jerde & Visscher, 2005). The ability to exclude a period that closely resembles search patterns could have the potential to reduce false‐positive periods of search, and we accounted for this as much as possible by removing locations in proximity to the colony as well as locations occurring at night before comparing methods. While not directly assigning a rest period, it is important to note that speed–tortuosity thresholds could be adapted to include the annotation of rest and travel, as well as specific search behavior. In a similar fashion, machine learning protocols could also be applied to predict behaviors other than diving.

Careful choices must be made in the selection and application of behavioral classification methods when inferring foraging. While all methods tested generally supported the hypothesis that search behavior leads to prey encounter and subsequent prey capture attempts in a wide‐ranging pelagic predator, there was considerable variation in the degree to which this was noted. The HMM method produced estimates of foraging behavior that most effectively encapsulated both search and prey capture components of foraging. As such, it would seem a sensible recommendation that HMM be used when identifying foraging (including both search and prey capture) areas is a priority. Across methods, rates of false negatives (dives occurring outside of search behavior) ranged from 19% to 70%. While some of this may be attributed to opportunistic feeding outside of search behavior, methods with high rates of false negatives suggest that care should be taken when using behavioral classification methods. That animals spend considerable time actively searching for prey, while prey capture occurs largely outside of this activity seems improbable, and poor classification of behaviors can have implications when considering time–energy budgets and subsequent reproductive success or survival. Methods such as HMM, EMbC, and thresholds had the lowest rates of dives occurring outside of search. These methods may be more attuned to capturing dive events and therefore represent a more inclusive definition of foraging, while FPT and *k*‐means clustering may be more general in their identification of search. Investigating the differences between methods may lead to increased understanding of the environmental cues used by predators to initiate search and prey capture as well as the scales at which these cues occur. Nevertheless, we reiterate the need for detailed exploratory analysis of movement data to prevent mis‐specification of behavior (Gurarie et al. (2016)) and argue for methods to be used based on suitability, and the questions being asked by researchers.

## CONFLICT OF INTEREST

None declared.

## AUTHOR CONTRIBUTIONS

AB, MJ, and SB conceived the initial ideas and designed methodology. WJG undertook analysis for HMM, AB undertook remaining analysis. MJ, WJG, EW, TWB, SCV, and KH provided advice and guidance on analytical frameworks and manuscript preparation. AB and MJ led the writing of the manuscript. All authors contributed critically to the drafts and gave final approval for publication.

## DATA ACCESSIBILITY

Data reported in this article are archived by Birdlife International (www.seabirdtracking.org).
