# Supplementary material for: Search and foraging behaviors from movement data: A comparison of methods
Source: Ecol Evol. 2017 Nov 23;8(1):13–24. doi: 10.1002/ece3.3593 (PMC5756868; doi:10.1002/ece3.3593)
Supplement: Supplementary file 1 [file ECE3-8-13-s001.docx]

**Electronic Supplementary Materials**

Below is a series maps to demonstrate the output of each method. Two random individuals were chosen to demonstrate the methods from each colony.

**
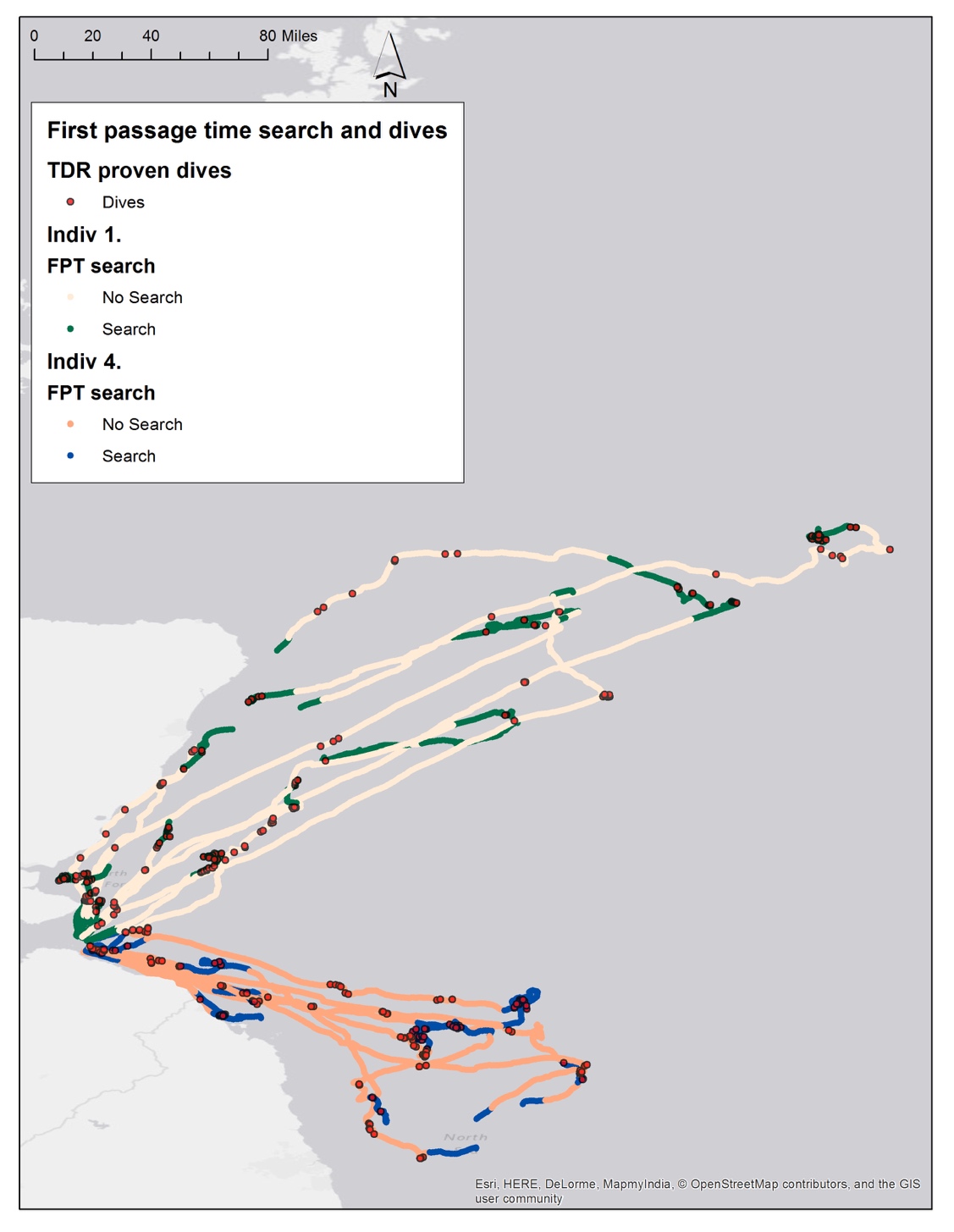
**

**Figure S1. Example first passage time analysis of two complete random gannet tracks (multiple trips in one tracking session) at Bass Rock. Locations close to the colony and nightime have been removed.**

**
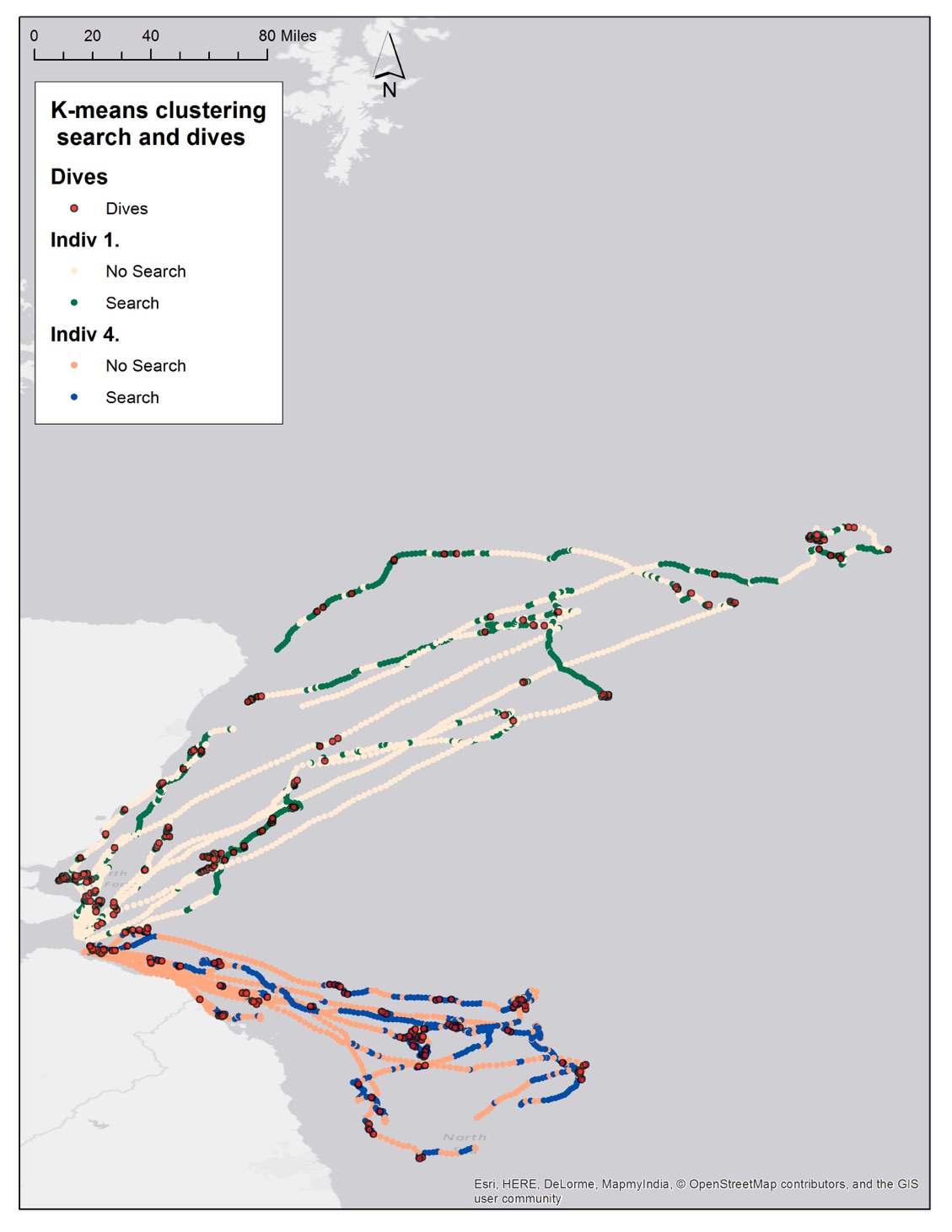
**

**Figure S2. Example K-means clustering analysis of two complete random gannet tracks (multiple trips in one tracking session) at Bass Rock. Locations close to the colony and nightime have been removed.**

**
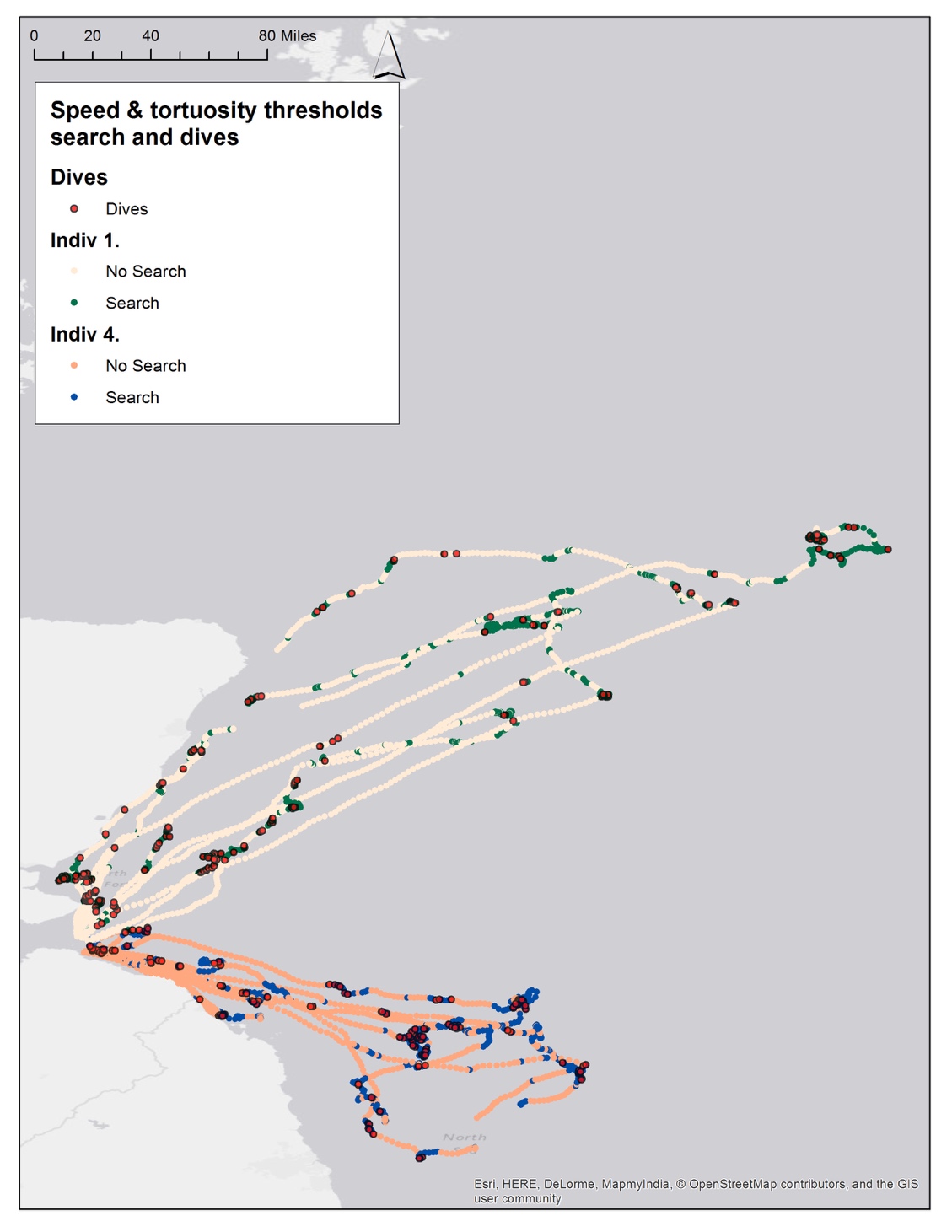
**

**Figure S3. Example speed & tortuosity thresholds analysis of two complete random gannet tracks (multiple trips in one tracking session) at Bass Rock. Locations close to the colony and nightime have been removed.**

**
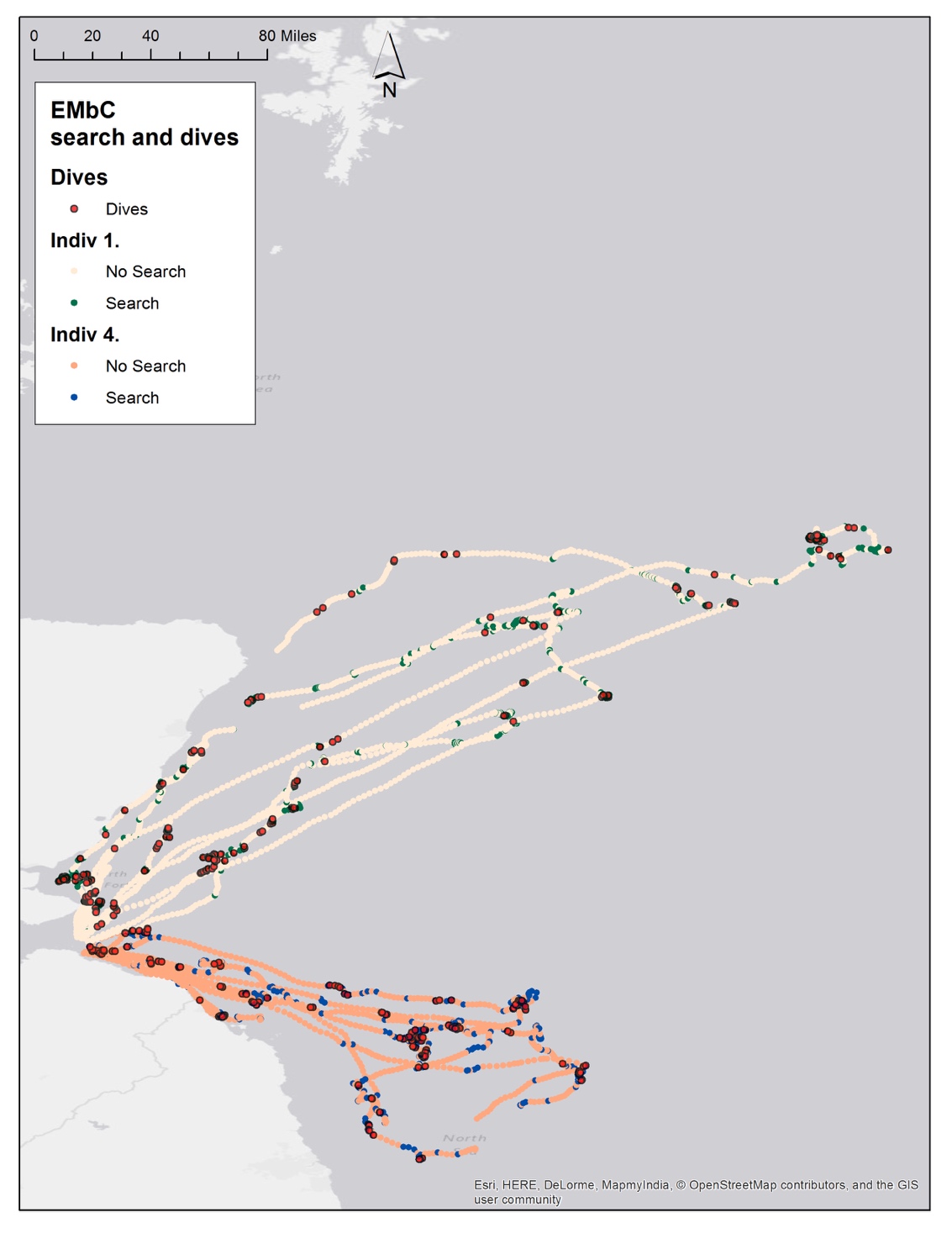
**

**Figure S4. Example expectation-maximisation binary clustering (EMbC) analysis of two complete random gannet tracks (multiple trips in one tracking session) at Bass Rock. Locations close to the colony and nightime have been removed.**

**
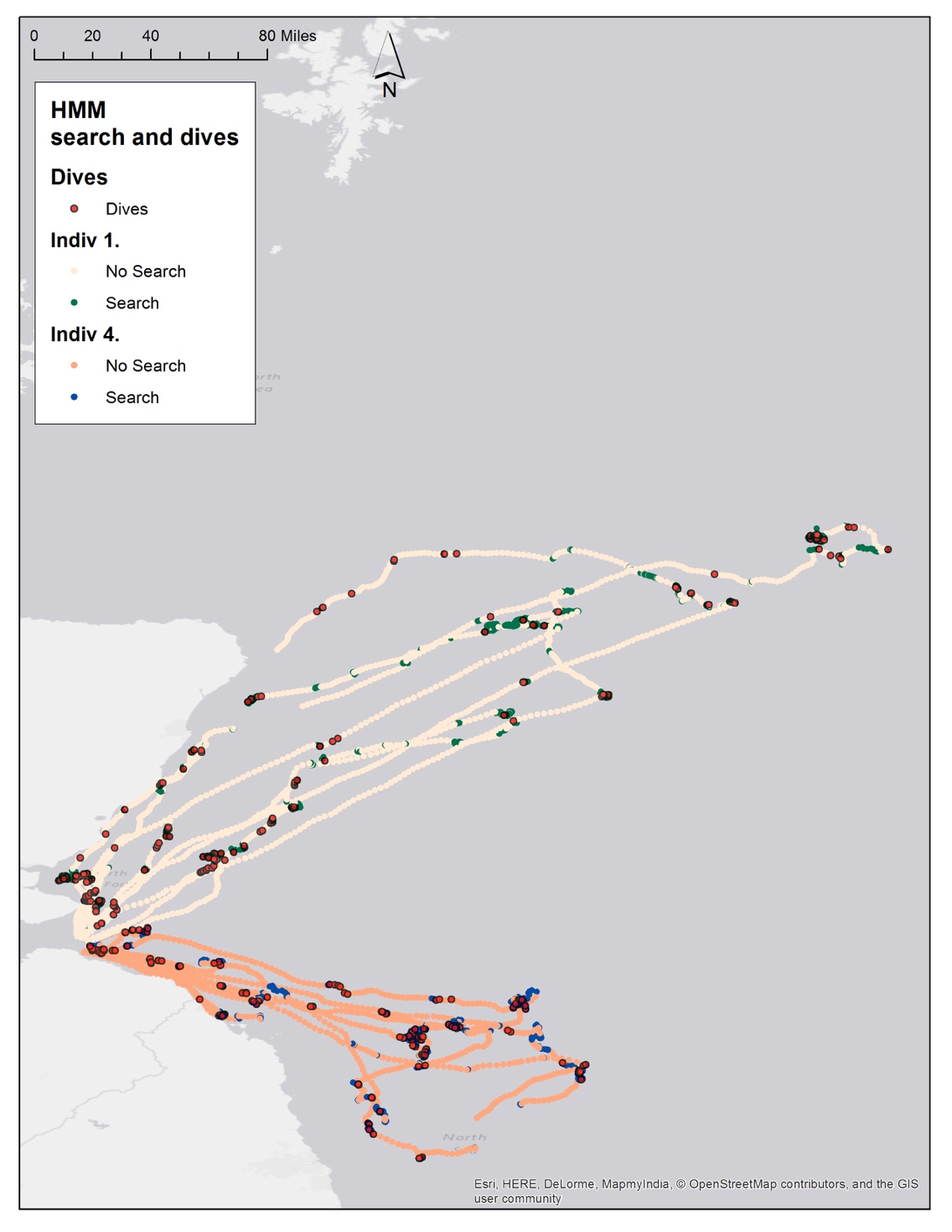
**

**Figure S5. Example hidden markov model (HMM) analysis of two complete random gannet tracks (multiple trips in one tracking session) at Bass Rock. Locations close to the colony and nightime have been removed.**

**
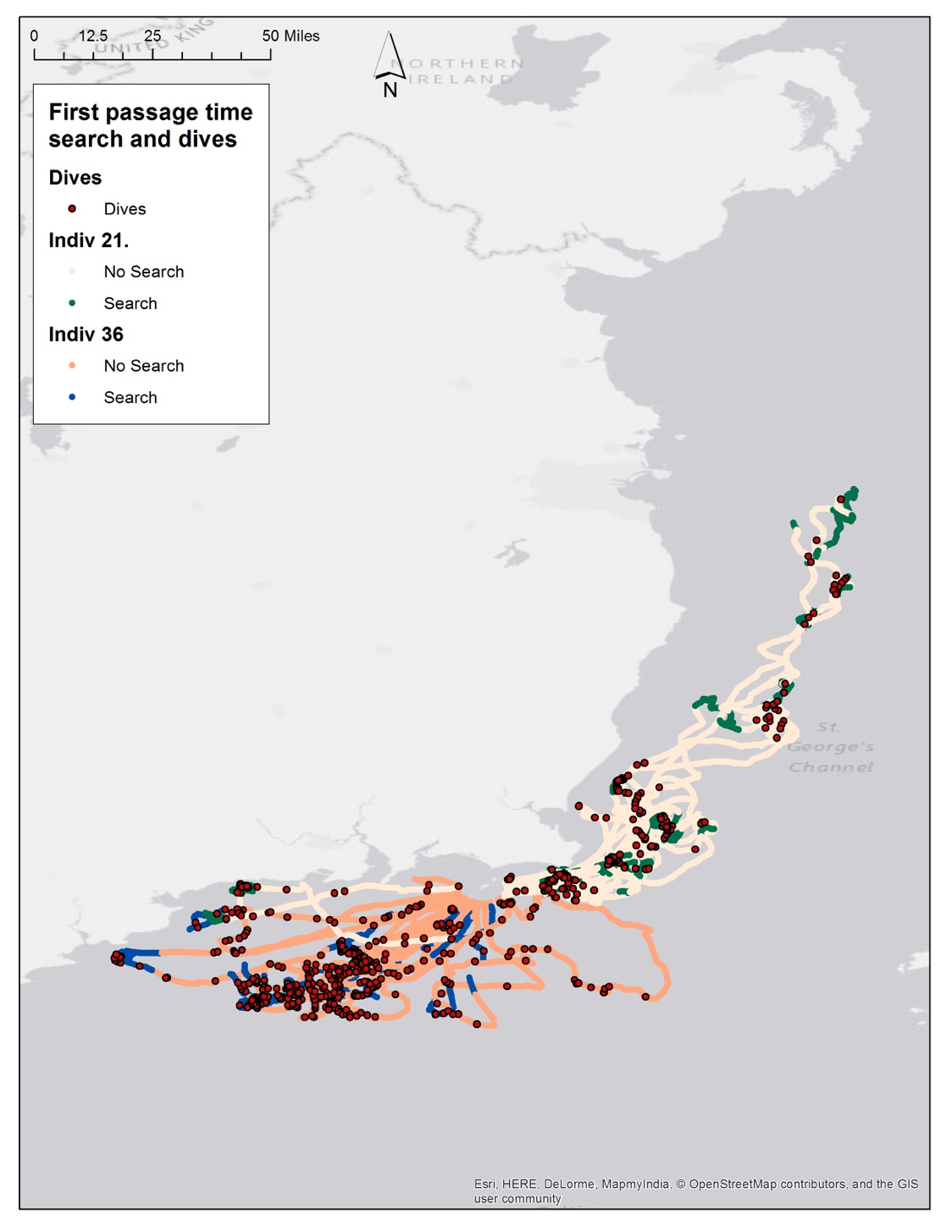
**

**Figure S6. Example first passage time analysis of two complete random gannet tracks (multiple trips in one tracking session) at Great Saltee. Locations close to the colony and nightime have been removed.**

**
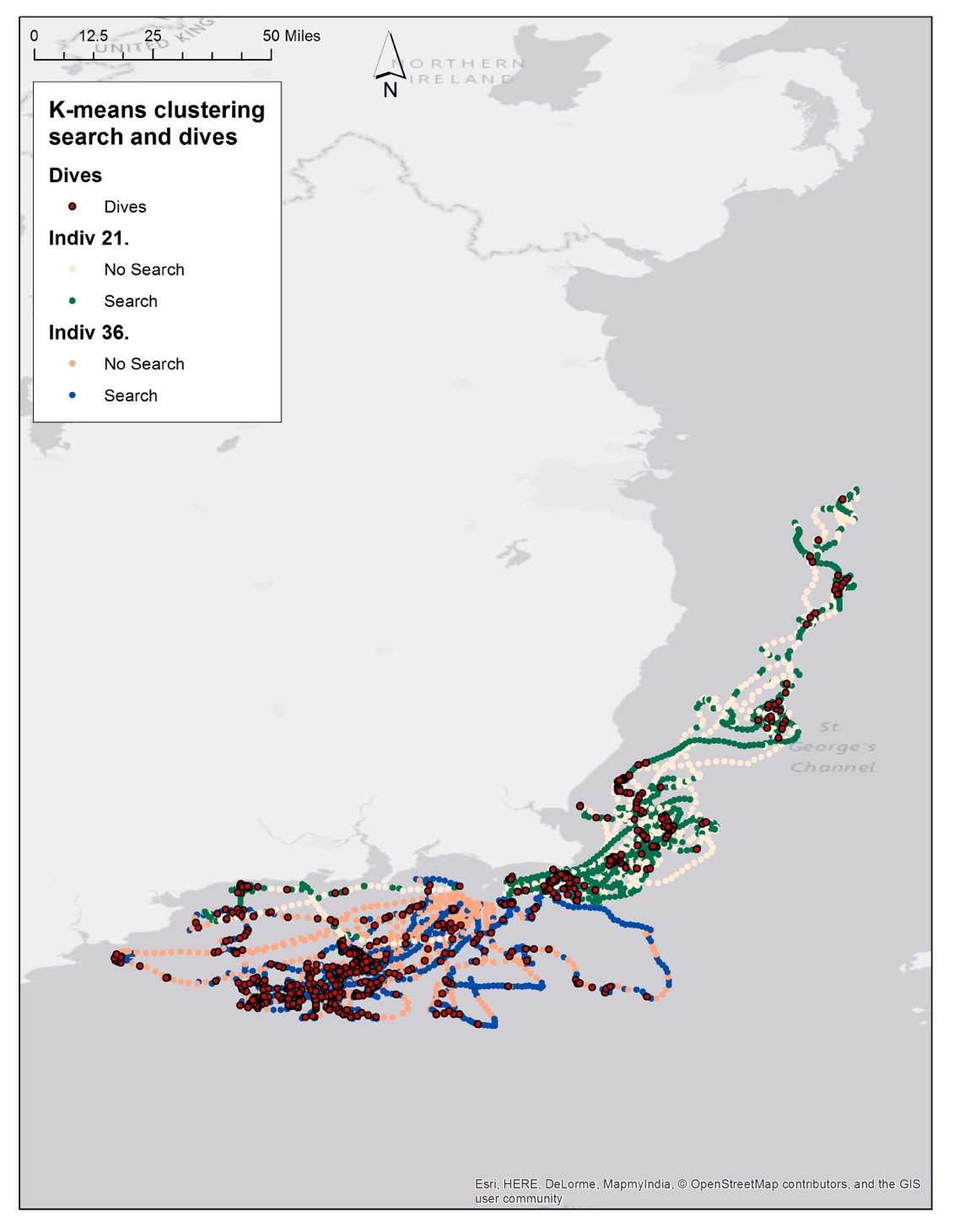
**

**Figure S7. Example K-means clustering analysis of two complete random gannet tracks (multiple trips in one tracking session) at Great Saltee. Locations close to the colony and nightime have been removed.**

**
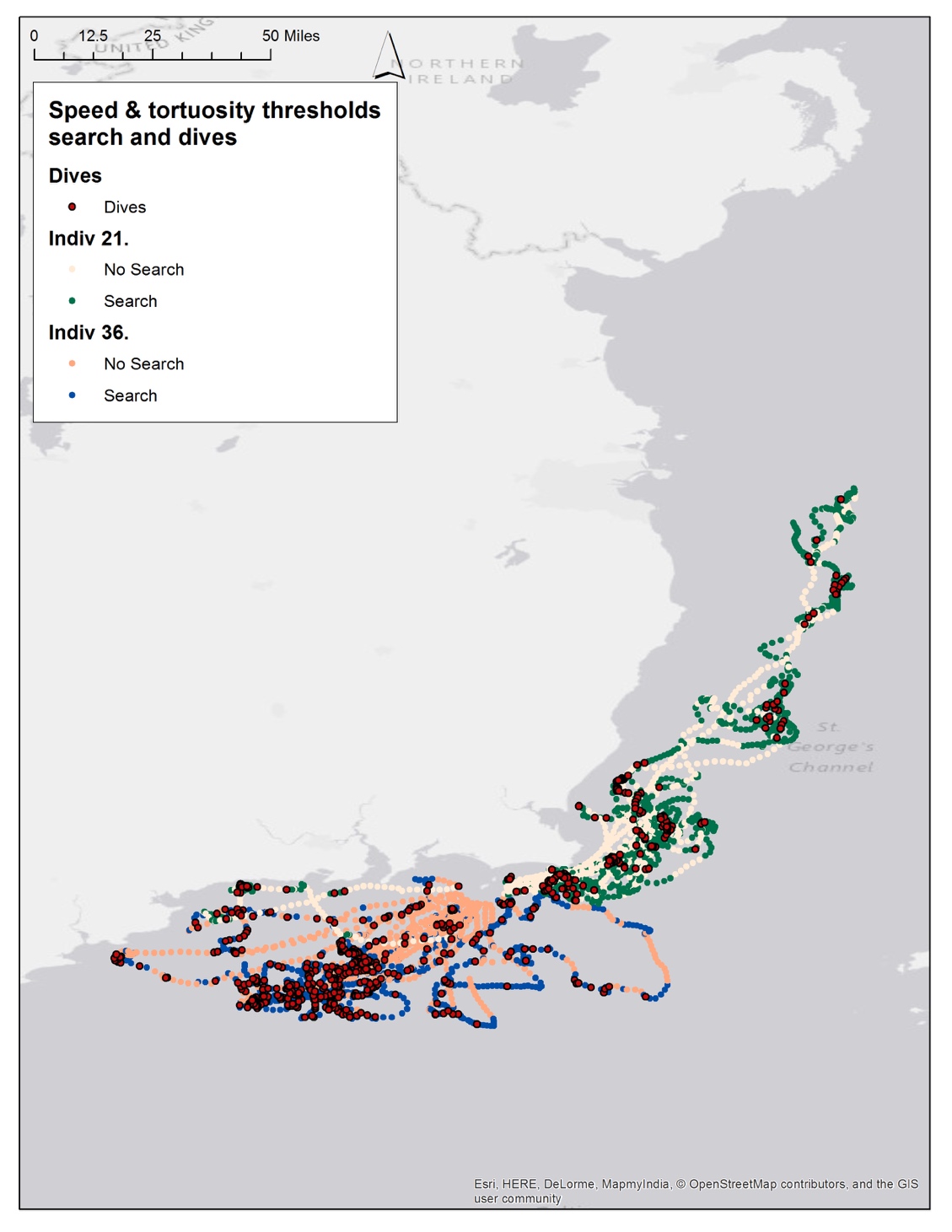
**

**Figure S8. Example speed & tortuosity thresholds analysis of two complete random gannet tracks (multiple trips in one tracking session) at Great Saltee. Locations close to the colony and nightime have been removed.**

**
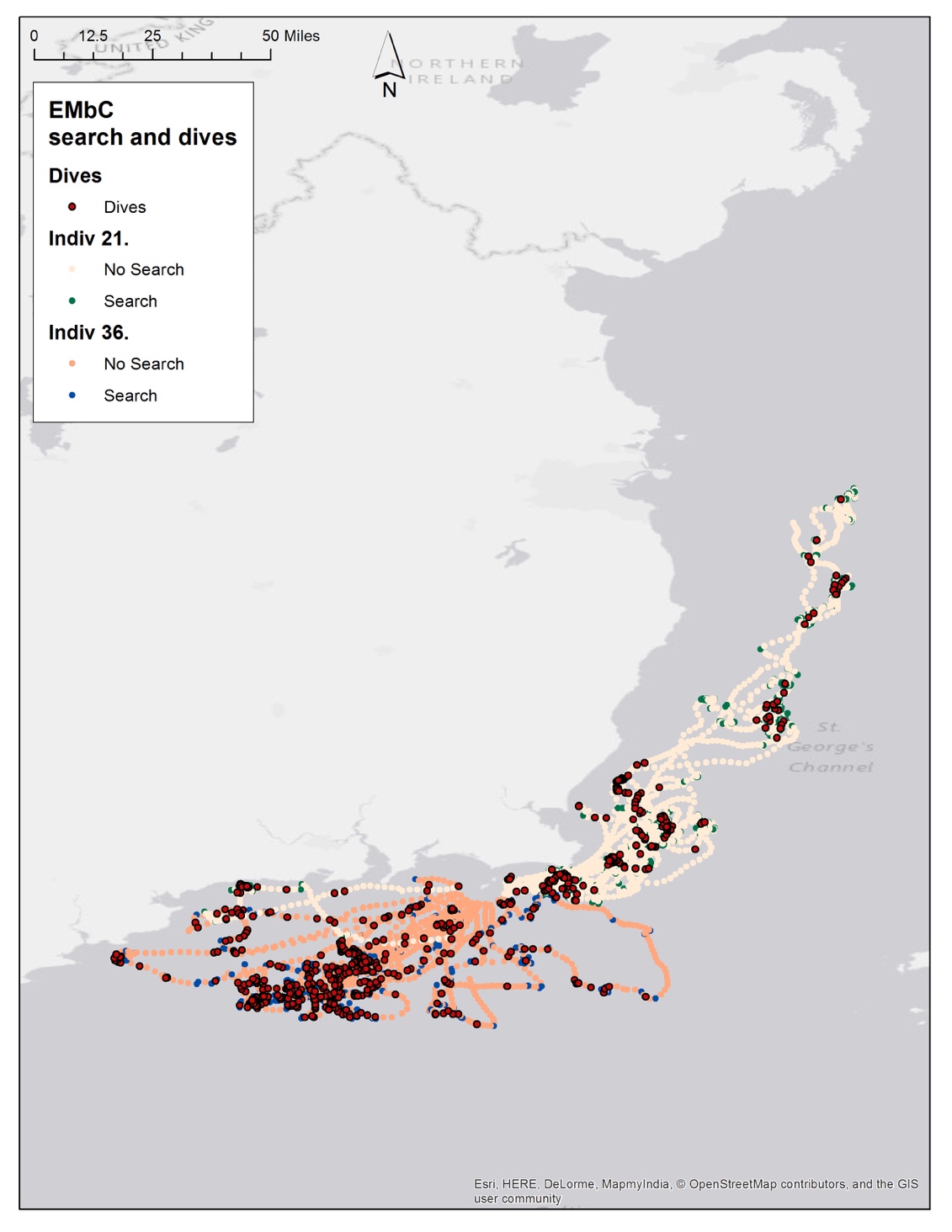
**

**Figure S9. Example expectation-maximisation binary clustering (EMbC) analysis of two complete random gannet tracks (multiple trips in one tracking session) at Great Saltee. Locations close to the colony and nightime have been removed.**

**
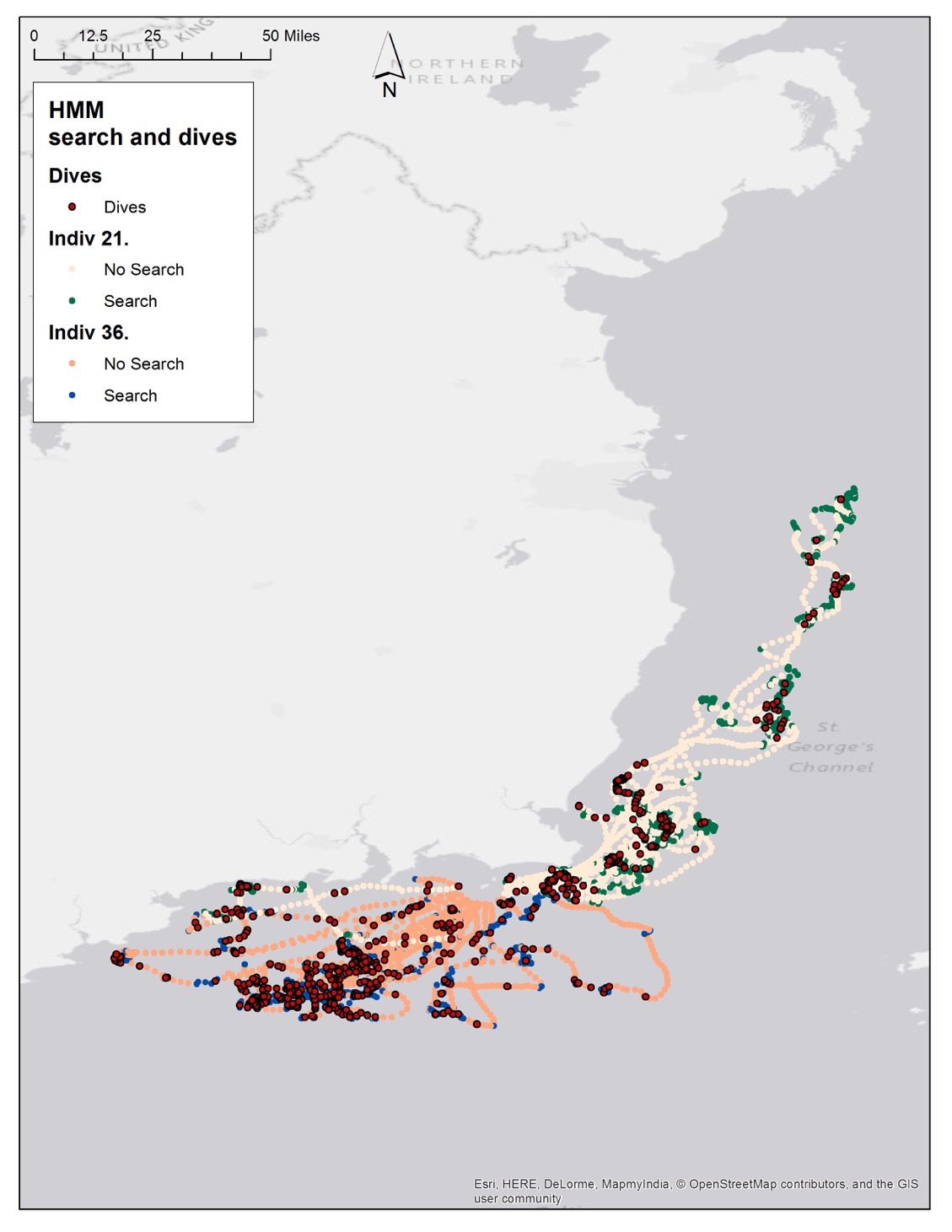
**

**Figure S10. Example hidden markov model (HMM) analysis of two complete random gannet tracks (multiple trips in one tracking session) at Great Saltee. Locations close to the colony and nightime have been removed.**
